# Ginsenosides in Modern Pharmaceutics: Mechanisms, Applications, Challenges, and Perspectives

**DOI:** 10.3390/biom16030350

**Published:** 2026-02-26

**Authors:** Mingyang Sun, Youduan Li, Ming Zhu, Haoming Luo, Ye Teng

**Affiliations:** School of Pharmacy, Changchun University of Chinese Medicine, 1035 Boshuo Road, Changchun 130117, China; smy0310622@163.com (M.S.); 18834110757@163.com (Y.L.); zhuming124@163.com (M.Z.)

**Keywords:** ginsenosides, molecular mechanisms, clinical application

## Abstract

Ginsenosides are the primary bioactive constituents of *Panax ginseng*, exhibiting multiple pharmacological activities, including neuroprotection, antitumor effects, anti-aging properties, and metabolic regulation. In this review, the molecular mechanisms of ginsenosides in treating neurodegenerative diseases, cancer, and metabolic disorders are summarized, and the current status of clinical translational research on ginsenosides in advanced gastric cancer, breast cancer, stroke, and diabetes is introduced, incorporating critical evidence regarding safety assessments and potential toxicity risks. In addition, recent advances in biotransformation and modern preparation technologies are reviewed. Innovative solutions, including nanodelivery systems, structural modifications, and AI-driven formulation design, are systematically discussed to address the current issues, such as low oral bioavailability and limited blood–brain barrier permeability. The future development of ginsenosides continues to face several critical challenges, including a scarcity of high-quality clinical evidence, an incomplete understanding of their mechanisms of action, a dearth of long-term safety data, and variations in quality between batches.

## 1. Introduction

Ginseng (*Panax ginseng* C. A. Meyer) has enjoyed a revered status in Traditional Chinese Medicine for more than 5000 years [1]. Ginseng is regarded as a precious medicinal plant in China, South Korea, Japan, and among Native Americans in North America, with its economic and medicinal value remaining timeless. With the development of scientific analytical techniques, ginsenosides have been isolated and are considered the key bioactive components in ginseng. Since their first isolation in 1963, more than 100 ginsenosides have been identified, and ongoing research continues to uncover new structures [2]. The structures of ginsenosides are highly related to their pharmacological effects, presenting diverse molecular targets and great therapeutic potential in health protection and disease treatment.

In recent years, the clinical application of ginsenosides has attracted considerable interest. Rare ginsenosides (RGs), which are named for their low or near-absent natural occurrence, such as Rg3, Rh2, and CK, exhibit increased bioactivity and clinical relevance, rendering them critical research targets in cancer adjuvant therapy and neuroprotection. However, their limited natural abundance and the complexity of extraction/conversion processes hindered their widespread clinical adoption, making RGs biotransformation and artificial processing an important area in current research. Several professional reviews on RGs immunomodulation [3], Rg3 delivery systems [4], gut microbiota regulation [5], and enzymatic conversion [6] have been published, mainly focusing on the single pharmacological effect of a specific ginsenoside or a particular technical aspect.

**Figure 1:**
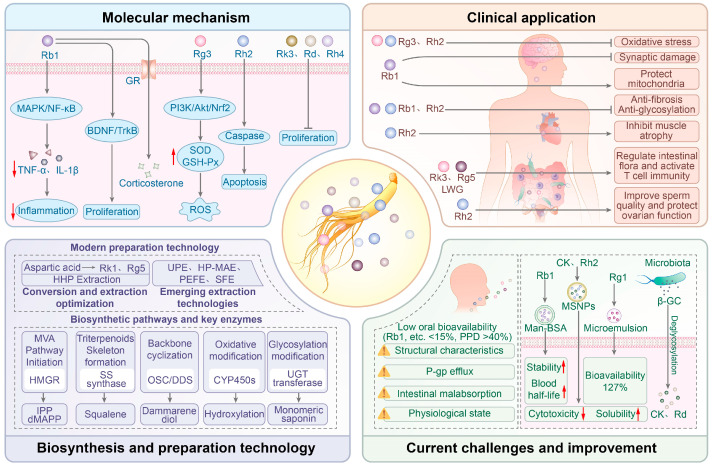
This review covers the molecular mechanisms, clinical applications, biosynthesis, current challenges, and improvements of ginsenosides.

In this review, the chemical characteristics, pharmacological mechanisms, and clinical applications of ginsenosides are systematically summarized. Figure 1 presents the main content of this review. A detailed overview of their mechanisms of action and application prospects in multiple fields, including neuroprotection, antitumor, anti-aging, and metabolic disease management, is provided. Current challenges in ginsenosides development, such as low ginsenoside content, poor bioavailability, and difficulties in clinical application, are discussed, and recent advancements in extraction, preparation, biosynthesis, and formulation optimization are introduced. Further in-depth mechanistic research and innovative improvements in the synthesis and delivery of ginsenosides, as a group of promising drug candidates, are expected in the future.

## 2. Chemical Characteristics of Ginsenosides

**Figure 2:**
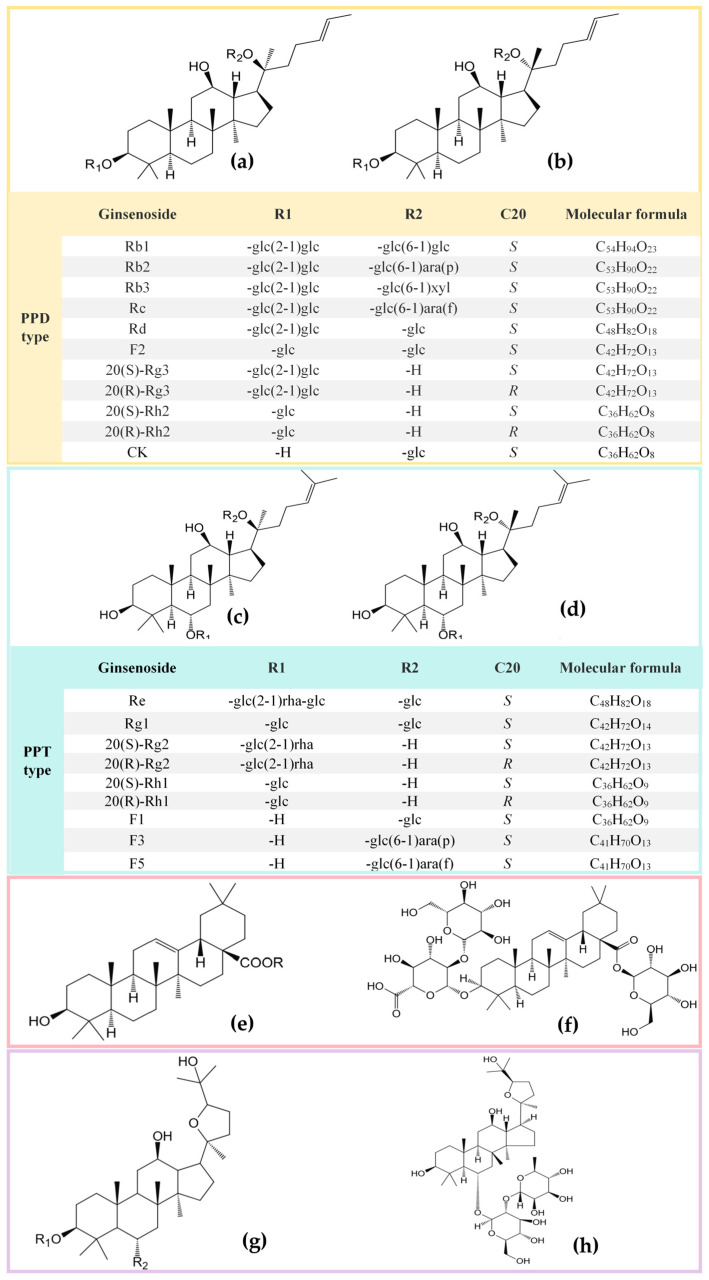
Ginsenosides are classified based on their aglycone skeleton. (**a**) Dammarane-type, 20(S)-PPD; (**b**) dammarane-type, 20(R)-PPD; (**c**) dammarane-type, 20(S)-PPT; (**d**) dammarane-type, 20(R)-PPT; (**e**) oleanane-type, OA; (**f**) Ro; (**g**) dammarane-type, OT; (**h**) pseudoginsenoside F11.

The structural diversity of ginsenosides is mainly a consequence of the high variety of sugar chains connected to different aglycone backbones [7]. In general, based on the structure of aglycone, ginsenosides can be classified into four primary types: protopanaxadiol (PPD), protopanaxatriol (PPT), oleanolic acid (OA), and ocotillol (OT) (Figure 2) [8,9]. The dammaranne type includes 20(S)-PPD and 20(S)-PPT, which share a four-ring hydrophobic steroid-like structure with sugar moieties but differ in carbohydrate moieties at C3, C6, and C20. In the PPD group, such as Rb1, Rb2, Rb3, Rc, Rd, Rg3, Rh2, and compound K, sugar residues are attached to the hydroxyl group at C-3 and/or C-20, while in the PPT group, such as Re, Rf, Rg1, Rg2, and Rh1, sugar moieties are attached to the hydroxyl group at C-6 and/or C-20 [8,9]. The OA type consists of a pentacyclic structure with an aglycone OA, such as ginsenoside Ro; the OT type has a tetracyclic structure with a tetrahydrofuran ring as a side chain, such as pseudoginsenoside F11 [10,11,12].

The structural characteristics of ginsenosides are very important for their classification and pharmacological effects. These characteristics offer a theoretical basis for the research and development of pharmaceuticals based on ginsenosides and explain why ginsenosides are widely used in various fields, such as neuroprotection, cancer, and immunomodulation [8,13,14]. For example, the antioxidant activity of PPD-type saponins is significantly stronger than that of PPT-type saponins, and their anti-fatigue effects also surpass those of the latter [15]. Research has also confirmed that the stereochemical configuration at the C-20 position exerts a decisive influence on activity, with 20(S)-Rg2 exhibiting a stronger inhibitory effect on catecholamine secretion in bovine adrenal chromaffin cells than 20(R)-Rg2 [16].

Among the numerous ginsenosides, RGs attract especially great interest due to their unique characteristics. RGs, such as CK, Rg3, and Rh2, are believed to be responsible for the pharmacological effects of ginseng [13,17]. RGs are synthesized in plant cells through the actions of specific glycosyltransferases, deoxygenases, and other enzymes. These RGs are obtained by adding uncommon sugar residues (L-rhamnose, D-fucose, D-arabinose, D-apiosyl, etc.) to the triterpenoid aglycone or by rare carboxylation, hydroxylation, and esterification [18]. RGs exist in extremely low natural concentrations (normally less than 0.1%) and are usually produced by partial hydrolysis of macro- or primary glycosides via steaming, acid/alkali treatment, or microbial metabolic transformation [13]. Overall, 144 RGs with promising clinical utility have been identified [8,13]. Significantly, various traditional treatments, including steaming [19], drying [20], acid hydrolysis [21], microbial degradation [22], and metal ion catalysis [23], were used to convert conventional ginsenosides into RGs, possibly improving the efficacy of ginseng. However, these methods had limitations. For example, high-temperature drying promotes oxidation and the Maillard reaction, leading to structural changes or decomposition of ginseng’s active components, including saponins [24]. Under acidic hydrolysis conditions, the reaction selectivity is poor, easily causing side reactions such as cyclization, dehydration, and isomerization of saponin molecules, resulting in high impurity levels and complex products [25]. Some microorganisms may produce harmful metabolic byproducts, such as biogenic amines and mycotoxins, which may not fully meet food or pharmaceutical safety standards and are associated with environmental issues [14,26]. Therefore, identifying how to safely and effectively obtain large quantities of RGs will be a key focus of future research.

## 3. Pharmacological Mechanism of Ginsenosides

We systematically searched Web of Science www.isiknowledge.com (accessed on 7 August 2025) for studies published from 2007 to 2025 using the keyword “ginsenosides” and analyzed them for active constituents, molecular targets, biomarkers and related signaling pathways. Ginsenosides exert their pharmacological activity through multiple signaling pathways, including the MAPK/NF-κB, PI3K/Akt, Nrf2, and hypothalamic–pituitary–adrenal (HPA) axis pathways, exhibiting broad pharmacological activities in neuroprotection (inhibiting neuroinflammation and oxidative stress), stress resistance (modulating the hypothalamic–pituitary–adrenal axis), antitumor effects (inducing apoptosis and inhibiting metastasis), and anti-aging (antioxidant effects and promoting collagen synthesis).

### 3.1. Neuroprotective Effects

Ginsenosides exert diverse effects on neuroprotection and stress regulation by influencing pathways related to neuroinflammation, oxidative stress, and neurotransmitter balance [27,28].

Ginsenoside Rb1 effectively suppressed microglial activation-induced neuroinflammation in the hippocampus by modulating the MAPK/NF-κB signaling pathway. Decreases in the levels of the pro-inflammatory cytokines IL-1β and TNF-α were observed in both mouse serum and brain tissue [27,28]. In a Sprague-Dawley rat model of Alzheimer’s disease, ginsenoside Rb1 reversed Aβ-induced synaptic damage by reducing reactive oxygen species levels in the hippocampus and suppressing the inflammatory cytokines TNF-α and IL-6. It was also found to promote microtubule stability, neuronal intracellular calcium homeostasis, and neuronal cell protection [28,29]. Additionally, ginsenoside Rb1 downregulated the expression of Beclin-1 and LC3-II by activating the PI3K/Akt pathway, thereby exerting neuroprotective effects against cerebral ischemia/reperfusion injury in mice [30]. In ten-week-old male C57BL/6 mice with PD model, ginsenoside Rb1 specifically inhibited α-synuclein aggregation by upregulating the astrocytic glutamate transporter glutamate transporter-1 (GLT-1), thereby mitigating glutamate-induced excitotoxicity [28].

Ginsenosides are also a potential alternative therapy for neurologically debilitating diseases induced by chronic stress. Proteomics studies demonstrated that ginsenoside Re augmented mitochondrial complex I activity through the PI3K/Akt pathway, accounting for an increase of 34.6%, thereby mitigating compromised energy metabolism in dopaminergic neurons [31]. Ginsenoside Rg2 and ginsenoside Rh1 enhanced cholinergic function, thereby ameliorating memory and learning abilities in mice by increasing acetylcholine (ACh) levels and inhibiting acetylcholinesterase (AChE). Ginsenoside Rg2 downregulated the expressions of Aβ, amyloid precursor protein (APP), and NMDA receptor protein (NR1) upregulated by cerebral ischemia/reperfusion injury and improved cognitive function. Ren et al. discussed the possible mechanisms underlying the antidepressant effect of ginsenoside Rg2 in a chronic mild stress mouse model [32]. Compared with the model group, Rg2 administered with fluoxetine for two weeks consecutively completely reversed depression-like behaviors in mice modeled with chronic mild stress for two weeks. Furthermore, protein blotting assay results showed that Rg2 upregulated the protein expression of the brain-derived neurotrophic factor (BDNF) signaling pathway in the mouse hippocampus. By injecting trkB shRNA into mice, the antidepressant effect of Rg2 was abolished, further demonstrating BDNF signaling pathway regulation by Rg2.

Furthermore, ginsenoside Rg1 exerts neuroprotective and anti-apoptotic effects in various models, including chronic stress, ischemia/reperfusion injury, and combined mannitol treatment, by regulating cAMP response element binding protein (CREB) phosphorylation/BDNF expression, p38/JNK, and PERK-eIF2-α-ATF4 signaling pathways. Ginsenoside Rg1 reversed the chronic stress-induced reduction in dendritic spine density by modulating the CREB phosphorylation signaling pathway; the p-CREB/CREB ratio increased 2.3-fold, and BDNF expression was significantly enhanced in the nucleus accumbens (*p* < 0.01) [33,34]. Ginsenoside Rg1 protected neural stem cells in an ischemia/reperfusion injury model from oxygen–glucose deprivation (OGD)-induced apoptosis and oxidative damage by suppressing p38/JNK phosphorylation [35]. In a rat model of cerebral ischemia, ginsenoside Rg1 and mannitol combined to prevent neuronal apoptosis by modulating the PERK-eIF2-α-ATF4 signaling pathway [36].

### 3.2. Anti-Stress Effects

Ginsenosides showed promising potential in neuroprotection and stress regulation with a multitarget synergistic mechanism [27,28]. The antidepressive effects of Rg1 were first analyzed in mice. Ginsenoside Rg1 relieved depressive symptoms and sleep abnormalities by modulating corticosterone levels and upregulating glucocorticoid and androgen receptor expression in the prefrontal cortex and hippocampus [37]. The antidepressant effect of ginsenoside Rg1 was possibly related to the modulation of the HPA and hypothalamic–pituitary–gonadal (HPG) axes [37], which are the core neuroendocrine axes mediating stress adaptation [38]. Ginsenoside-enriched ginseng extracts were also demonstrated to modulate the HPA axis by increasing glucocorticoid receptor levels in mice, decreasing FKBP51 expression, and improving negative feedback regulation [39], subsequently alleviating depressive behaviors in mice [39]. Ginsenoside Rg1 increased the expression of glucocorticoid receptor in the frontal cortex and hippocampus of mice, thereby protecting neurons from glucocorticoid-induced damage and neuroinflammation and indicating the neuroprotective and stress-modulating effects of Rg1 on the HPA axis [40].

### 3.3. Antitumor Effects

Ginsenosides Rh2, Rg3, Rk3, Rd, and Rh4 demonstrated antitumor activity through cancer cell growth inhibition [41,42,43], cell death induction [42], blood vessel formation obstruction [42], metastasis suppression [42], and immune response regulation [44].

Ginsenoside Rh2 was used widely in preclinical cancer research because of its potent antitumor effects and low toxicity. Ginsenoside Rh2 showed efficacy against various cancers, including breast [45], acute lymphoblastic leukemia (T-ALL), endometrial, non-small-cell lung [41,46], pancreatic [47], prostate [48], and CRC [49]. The primary anticancer mechanism of Rh2 involved activating the caspase pathway, upregulating pro-apoptotic proteins such as Bax, and downregulating anti-apoptotic proteins such as Bcl-2, leading to cancer cell apoptosis.

Ginsenoside Rg3 is a significant ginsenoside with potent antitumor effects caused by inhibiting human colon cancer cells (HCT116) proliferation through G1 phase cell cycle arrest and suppressing key signaling pathways, such as the PI3K/Akt and NF-κB pathways. Additionally, Rg3 hindered metastasis in mouse melanoma cells (B16-BL6) by suppressing matrix metalloproteinases (MMPs) and inhibited angiogenesis by downregulating vascular endothelial growth factor (VEGF), thereby limiting blood supply to lung squamous cell carcinoma cells (SK-MES-1) [50,51].

RGs, such as ginsenosides Rk3, Rd, and Rh4, exhibit significant anticancer effects. Ginsenoside Rk3 inhibited the growth of H460 and A549 non-small-cell lung cancer (NSCLC) cell lines, induced apoptosis, and inhibited angiogenesis [46]. Ginsenoside Rd reduced the spread of colorectal cancer in human colorectal cancer cell lines and in a colorectal cancer metastasis mouse model [52]. Ginsenoside Rh4 exhibited anti-metastatic activity against lung adenocarcinoma by inhibiting the JAK2/STAT3 signaling pathway in both A549 and PC9 cell lines, as well as in an A549 xenograft tumor model [53].

Long-term toxicological studies have confirmed that ginsenosides exhibit low toxicity in five-month-old female C57BL/6J mice, particularly in non-cancerous tissues [50], establishing a foundation for their potential clinical use. Combining ginsenosides with other treatments, such as transcatheter arterial chemoembolization (TACE) or chemotherapy, could help to better manage tumors, prolong the survival of patients, and alleviate treatment side effects [54]. Ginsenoside Rg3 was also advantageous for NSCLC patients in terms of alleviating symptoms, myelosuppression, and prolonging survival [55,56,57]. According to Peng et al., the synergistic effects of ginsenoside Rg3 with conventional chemotherapy in advanced NSCLC were improved remission rate, disease control, and overall survival of patients [55]. Meanwhile, the combined application of ginsenoside Rg3 with oxaliplatin exhibited anticancer effects by inhibiting SMMC-7721 cells proliferation and promoting apoptosis by suppressing PCNA and Cyclin D1 expression [58]. Low-polarity ginsenosides (LWGs) exhibit immunomodulatory activity, with their mechanism potentially enhancing immune regulation by modulating the gut microbiota [59]. Notably, immunomodulatory effects of LWGs in colorectal cancer were significantly manifested in enhancing T-cell activation and promoting synergistic interactions with CRC treatments [60]. The combination of immunotherapy and conventional chemotherapies in gastrointestinal cancers was reportedly synergistic, possibly by modulating chemoresistance with mechanisms involving drug transporters and modulating the tumor microenvironment [61,62]. However, further clinical trials are necessary to standardize their dosages and validate their potential in human cancer therapy.

### 3.4. Anti-Aging Effects

Oxidative stress played a key role in lifestyle-related disease progression and was linked to the balance between producing and removing reactive oxygen species (ROS), which were closely associated with human aging [63].

The antioxidant properties of ginseng relied primarily on PPD (e.g., Rb1) and PPT (e.g., Rg1, Re) saponins, which reduced intracellular ROS levels and improved calcium homeostasis, thereby mitigating oxidative stress [64,65,66,67,68]. The significant antioxidant capacity of ginsenoside Rg1 has been demonstrated in antioxidant studies. For example, ginsenoside Rg1 notably suppressed apoptosis and cystatin inhibitor-3 activation and decreased ROS and malondialdehyde (MDA) production [69,70]. Moreover, in SH-SY5Y cells, ginsenosides regulated the Nrf2-ARE signaling pathway and upregulated the expression of antioxidant enzymes such as heme oxygenase-1 (HO-1) and glutathione S-transferase (GST) [65,71].

Ginsenoside also appeared in the research focused on skin hydration and collagen synthesis. Ginsenoside Rb1 was observed to increase hyaluronic acid production in human keratinocytes via the ERK/AKT signaling pathway, thereby improving skin hydration and augmenting collagen synthesis [72,73]. In in vitro human immortalized epidermal cells (HaCaT) and HDF models, as well as in the in vivo model of UV-irradiated hairless mice, Rb1 was also demonstrated to exert anti-aging effects on the skin by promoting type I collagen synthesis and inhibiting UV-induced apoptosis [74,75]. In addition, CK topical application increased the hyaluronic acid content in the skin of hairless mice [76]. Furthermore, in UV-irradiated NHDF cells, ginsenoside Rg3 stimulated collagen synthesis and suppressed the expression of matrix metalloproteinase-1 (MMP-1), thereby effectively preventing skin aging and photo-aging [77,78].

Ginsenoside Rg3 was applied in nutraceuticals owing to its anti-aging effects on metabolism [79,80], immune function [64,79,81], mitochondrial activity, and stem cell proliferation [64,79,81]. Rg3 was reported to protect cellular homeostasis by modulating the gut microbiota, telomerase activity, and mitochondrial function [64,79,81] while simultaneously upregulating the expression of anti-inflammatory factors (e.g., IL-10) and decreasing the expression of pro-inflammatory factors (e.g., IL-6 and TNF-α) [64,79,81]. These results provided new perspectives for treating age-related diseases and maintaining health.

### 3.5. Metabolic Research

Metabolic research is a critical step in developing new drugs. Optimizing the metabolic stability of lead compounds can improve their pharmacokinetic and pharmacodynamic properties. Simultaneously, identifying active metabolites aids in discovering new chemical entities and enables early exclusion of drug candidates with potential toxic metabolic risks [82]. Herein, the metabolic targets and regulatory mechanisms within ginsenoside metabolic reprogramming pathways are summarized in Table 1. These results can provide theoretical foundations and novel perspectives for drug development.

## 4. Clinical Application of Ginsenosides

Currently, research on the clinical application and translational potential of ginsenosides spans multiple therapeutic domains. Regarding intervention in neurodegenerative diseases, in a model that administered 1.4% isoflurane anesthesia to five-month-old female C57BL/6J mice, Rb1 exhibited synaptoprotective properties, reversed the synaptic dysfunction caused by isoflurane surgery, and improved glutamatergic modulation [87]. In metabolic disorder management, based on its hormone-like effects and influence on metabolic processes, Rg3 increased GLP-1 secretion in L-cells and enhanced the insulin release and glucose tolerance in type 2 diabetic patients [83]. Ginsenosides Rg1 and Re act by expressing rat estrogen receptors to ameliorate menopausal symptoms and osteoporosis [88]. Table 2 lists completed clinical trials on ginsenosides, covering advanced gastric cancer [89,90], breast cancer [91], and stroke prevention [92], demonstrating that ginsenosides have yielded certain results in clinical applications. Although current experimental results indicate that ginsenosides exert positive effects through multiple mechanisms in clinical applications across various systems, positioning them as multifunctional potential therapeutic agents, their pathological impacts on these diseases warrant further investigation [93,94]. For example, in a 28-day repeated-dose toxicity study in rats, high doses of ginsenoside Rg2 significantly prolong prothrombin time and reduce total cholesterol levels, suggesting potential interference with coagulation function and lipid metabolism [95]. Additionally, ginsenosides Rg1 and Rg3 exhibit teratogenic effects in rat and mouse embryo culture, inducing abnormal embryo formation and impairing pre- and post-implantation development [95]. Further validation through clinical trials is necessary. Meanwhile, the oral bioavailability of ginsenosides and formulation stability issues still require further investigation. Overall, the oral bioavailability of most ginsenosides (Rb1, Rb2, Rd, Rg3, Re, etc.) is below 15% [96]. Regarding formulation stability, ginsenosides exhibit limited chemical stability in solution (e.g., Rg1 lipid formulations remain stable for up to 8 h) and are susceptible to degradation by gut microbiota, temperature fluctuations, and pH changes [97]. These factors make it challenging to maintain parent drug plasma concentrations [96]. Consequently, formulation improvements and stability optimization remain critical areas requiring further research.

## 5. Current Challenges and Improvements

### 5.1. Ginsenoside Biosynthesis and Preparation Technology

The industrial synthesis of RGs is constrained by their extremely low content in natural plants (typically <0.1%), complex structures, lengthy synthetic pathways, diverse raw material sources, and incomplete elucidation of key enzyme functions and regulatory mechanisms. Traditional extraction methods fail to achieve the required purity, making the development of novel preparation methods crucial for enhancing overall yield and purity. Research indicates that modifying the expression levels of certain enzymes can significantly impact the final saponin yield. For example, overexpressing the PgFPPS gene further enhanced the saponin content in ginseng hairy roots 2.4-fold more than the control, while RNA interference analysis showed that suppressing the PgSQE1 gene could decrease saponin biosynthesis and increase phytosterol accumulation [102,103]. In addition, further increasing the expression of their multifunctional duplicated genes, PgSQE2, could compensate for the metabolic defects caused by RNA interference of the PgSQE1 gene and lead to functional redundancy in plant metabolic control [102,103]. Researchers have also employed CRISPR/Cas9-mediated miRNA modulation to increase saponin synthase (CYP450) expression and inhibit degradation pathways, resulting in enhanced ginsenoside production [102]. Ginsenoside biosynthesis involves a complex network of enzymes and metabolic pathways, providing excellent targets for metabolic engineering. By integrating multi-omics data and advanced biotechnologies, we can produce specific ginsenosides at desired levels [104,105,106]. Research has also confirmed ginsenosides Rg3 and Rh2 using chemical hydrolysis and microbial enzymatic methods to modify glycosyl structures [85,107,108]. At 110 °C, aspartic acid can convert RGs from American ginseng into ginsenosides Rk1 and Rg5 [109]. In addition, ginsenoside Rg1 and Rb1 extraction increases with increased parameters in enzyme-promoted high hydrostatic pressure (HHP) extraction [110,111]. Modern extractions have remarkable benefits compared to classic Soxhlet extraction and thermal reflux. Usual enhancements attained by new extraction methods, such as ultrahigh-pressure extraction (UPE) [112], high-pressure microwave-assisted extraction (HP-MAE) [113], supercritical fluid extraction (SFE) [114], and pulsed electric field extraction (PEF) [115], include a shortened extraction time, reduced solvent consumption, and ease of automation [111,116]. For example, HP-MAE could obtain target compounds with high extraction efficiency of RGs in 70% ethanol solution within only 10 min, and the result was remarkably higher than that of Soxhlet and ultrasonic-assisted extraction methods [22]. Furthermore, ultrahigh-pressure extraction was preferred in a 50% ethanol solution at 500 MPA pressure [117]. By optimizing process parameters, modern technology can enhance RGs yield and provide a reference for future innovations in RG preparation. Therefore, systematic analysis of synthetic pathways and regulatory networks, structural and functional modification of core enzymes, and the development of efficient synthetic methods will become key directions for future advancement.

### 5.2. Ginsenoside Bioavailability and Delivery Strategies

The clinical application of ginsenosides is constrained by their low oral bioavailability and limitations in administration methods. Research indicates that the oral bioavailability levels of ginsenosides Rb1, Rb2, Rb3, Rd, Rg3, Rh2, CK, Re, and Rg1 are all below 15%, while only ginsenoside PPD exhibits an oral bioavailability exceeding 40% [96]. The oral bioavailability of ginsenoside Rb1 is only 4.35% [118,119]. The primary factors contributing to these limitations are the ginsenoside structure, P-gp efflux, and intestinal malabsorption. These factors result in poor pharmacokinetic properties of ginsenosides, hindering their further evaluation in clinical settings.

From a ginsenoside structural perspective, the dammarane skeleton confers poor hydrophilicity, while the sugar moiety reduces lipophilicity, resulting in limited solubility in most solvents [96]. According to the Bioequivalence Classification System (BCS), Rg1, Rb1, and others are classified as BCS Class III (high solubility, low permeability), while Rh2 is classified as BCS Class IV (low solubility, low permeability). Regarding P-gp efflux factors, studies indicate that P-gp activation reduces ginsenoside absorption [96]. Verapamil and cyclosporine A (P-gp inhibitors) decrease the ginsenoside efflux ratio, suggesting P-gp involvement in their efflux. Based on an analysis of intestinal absorption factors [120,121], it is well established that physical conditions influence metabolic processes. Ginsenoside oral bioavailability is affected by physiological states, such as dietary interventions or diseases. Reports confirm that conditions including high-fat diets, diabetes, cancer, depression, and AD all impact ginsenoside oral bioavailability [122]. Different health states lead to distinct metabolic states, and varying metabolic states result in different gut microbiota compositions. Gut microbiota may regulate ginsenoside oral bioavailability through three mechanisms: First, gut microbiota may release ginsenosides bound to (or captured by) food/drug matrices (e.g., fiber, protein), thereby increasing free ginsenoside levels. Second, the gut microbiota may regulate ginsenoside metabolic processes (e.g., deglycosylation) [122,123,124]. Third, the gut microbiota may modulate intestinal permeability [96].

To overcome these limitations, researchers have developed various ginsenoside delivery systems. Specifically, nanotechnology greatly enhanced ginsenoside Rb1 oral bioavailability using Man-BSA@Rb1 NPs; the encapsulation efficiency was 96.7% [125]. Self-assembled nanoparticles, such as ginsenoside Rb1 combined with betulinic acid, form stable particles of about 100 nm, prolonging blood half-life and enhancing tumor targeting [126]. Singh et al. [127] loaded CK and Rh2 onto MSNPs and reported increased cytotoxicity against various cell lines and better anti-inflammatory effects in RAW264.7 cells. The mesoporous structure of MSNPs protects drugs, enhancing drug efficacy based on sustained release and high loading capacity. A microemulsion system increases ginsenoside Rg1 bioavailability by 127% based on improved solubility and permeability [128]. The intestinal flora modulates ginsenoside Rb1 deglycosylation through β-glucosidase, yielding active metabolites such as ginsenosides Rd and CK. High ginsenoside Rb1 concentrations impact galactose metabolism, altering drug absorption by modifying intestinal carbohydrate metabolism via glycosidases such as EC 3.2.1.22 [129,130,131]. In addition to summarizing the aforementioned studies, we have also categorized existing ginsenoside delivery systems. These primarily include nanoemulsion/microemulsion systems [128], liposomes [128], polymeric nanoparticles [3], solid lipid nanoparticles [3], nanostructured lipid carriers [128], and self-emulsifying delivery systems. Compared to traditional delivery systems, such as conventional tablets/capsules [132], standard hydrogels, microspheres, and cyclodextrin inclusion complexes [133,134,135], these technologies offer distinct advantages for ginsenoside delivery. Table 3 below compares traditional drug delivery methods with existing ginsenoside delivery approaches, analyzing their respective strengths and limitations. Although progress has been made in enhancing ginsenoside oral bioavailability and improving delivery methods through nanotechnology-based modifications and structural optimization, stability and studies on the potential toxicity of long-term in vivo metabolism of nanomedicines remain critical challenges [126,127].

At the synthetic biology level, although *Saccharomyces cerevisiae* cell factories have elevated ginsenoside Rh2 titer to 2252.3 mg/L through multi-module metabolic engineering strategies (including heterologous gene expression, metabolic flux optimization, and protein scaffold construction) [86], the catalytic efficiency and substrate specificity of key enzymes (such as CYP450 oxidases and UGT glycosyltransferases) remain bottlenecks limiting non-natural ginsenoside large-scale production (e.g., dammarene-2,2-diol-type saponins). Recently, by introducing a codon-optimized gene via the CRISPR/Cas9 system combined with endoplasmic reticulum engineering, efficient synthesis of the non-natural saponin 3β-O-Glc2-DM (766.3 mg/L) was achieved. This derivative demonstrated protective activity against hypoxia/reoxygenation injury in cardiomyocytes [137]. Clinically, significant interindividual variability in microbiota-mediated deglycosylation metabolism leads to inconsistent ginsenoside oral bioavailability and therapeutic efficacy. Future research should establish personalized dosing strategies based on gut microbiota profiling and reduce P-gp efflux ratios while enhancing membrane permeability through structural modifications (e.g., octyl esterification or sulfation) [138]. Therefore, constructing a large-scale multidimensional database encompassing structure, metabolism, and clinical endpoints, and integrating organ-on-a-chip with single-cell omics technologies, will be the key to overcoming existing translational bottlenecks and realizing precision medicine applications.

## 6. Conclusions

As multitarget natural bioactive molecules, systematic advances have been achieved in elucidating the molecular mechanisms of ginsenosides. Existing research has clarified that they regulate key enzyme systems and multiple signaling pathways. Furthermore, the preliminary elucidation of their multi-component synergistic mechanisms and gut microbiota-mediated metabolic activation pathways has further expanded precision intervention strategies based on ginsenosides. These achievements not only establish the scientific value of ginsenosides in disease prevention and treatment but also provide a crucial library of candidate compounds and validated targets for developing innovative drugs targeting neurodegenerative diseases, metabolic syndrome, and malignant tumors, demonstrating clear potential for clinical translation. In addition, significant progress has been made in recent years regarding ginsenoside isolation and purification, their pharmacological mechanisms, and delivery systems. Particularly in the field of biosynthesis, partial synthetic pathways have been successfully deciphered by leveraging metabolic engineering and synthetic biology strategies. This provides a viable technical pathway to overcome plant-source limitations and achieve heterologous synthesis of RGs.

Although preclinical studies have demonstrated the therapeutic potential of ginsenosides, their translation into clinical practice encounters substantial challenges. The primary limitation stems from a systematic lack of translational evidence: current research is predominantly confined to in vitro and animal models, lacking large-scale, multicenter randomized controlled trials that adhere to international standards. Additionally, existing clinical studies suffer from limited sample sizes and inconsistent trial protocols, which impede the generation of high-quality evidence-based medical proof. Moreover, low ginsenoside bioavailability (with oral bioavailability below 15% for certain components) and their intricate pharmacokinetic properties, along with variations in chemical composition across batches due to differences in raw material sources, processing techniques, and extraction methods, collectively pose significant barriers to achieving consistent therapeutic efficacy. Future research should prioritize establishing a standardized quality control system based on chemical fingerprinting and developing standardized testing and evaluation methods for various ginsenosides and their metabolites. Simultaneously, there is an urgent need to conduct multicenter randomized controlled clinical trials in oncology, cardiovascular diseases, and metabolic disorders while devising personalized dosing strategies based on gut microbiota profiling to address issues of therapeutic heterogeneity and inadequate safety evaluations.

The primary challenges in ginsenoside applications revolve around the present deficiencies in systematic evaluations concerning formulation safety and long-term toxicity risks. Although acute toxicity studies demonstrated relative safety at conventional doses (LD50 > 2000 mg/kg), recent 90-day subchronic toxicity studies revealed that high doses (≥600 mg/kg) of RGs mixtures can induce gut microbiota dysbiosis, elevated liver enzyme levels (ALT, LDH, AKP), and abnormalities in vitamin B6 metabolic pathways. This suggests potential risks of hepatotoxicity and intestinal barrier damage upon prolonged exposure. Additionally, concerns remain regarding the long-term in vivo behavior of nanodelivery systems (e.g., accumulation of PLGA degradation products, sustained activation of the reticuloendothelial system), the potential carcinogenic/mutagenic properties of byproducts generated during processing (e.g., sulfur fumigation derivatives), the mutagenicity of such byproducts, the induction effects of ginsenosides on the CYP450 enzyme system (particularly CYP1A2), and the risk of interactions with anticoagulants (e.g., warfarin), all of which have not been thoroughly evaluated in large-scale pharmacovigilance studies.

Interestingly, integrating artificial intelligence with novel delivery technologies presents transformative opportunities to address these challenges. In the targeted delivery field, AI-driven formulation design has achieved over 99% accuracy in predicting nanoparticle performance, significantly enhancing drug delivery efficiency and stability. Magnetic molecularly imprinted polymer (MMIP) nanoparticles with preprogrammed recognition capabilities have demonstrated outstanding performance in extracting the target component ginsenoside Rb1 and protecting myocardial cell mitochondrial function. Concurrently, novel formulation technologies, such as nanodelivery systems and solid dispersions, have been proven to significantly enhance drug absorption and tissue distribution, providing effective pathways to overcome bioavailability bottlenecks. Future efforts should focus on overcoming limitations in existing databases—such as limited data volume, insufficient diversity, and slow updates—by constructing multidimensional databases integrating chemical structures, metabolic characteristics, and clinical endpoints. Combined with the development of innovative delivery systems and the establishment of standardized quality evaluation systems, these approaches will propel ginsenosides toward precision medicine applications.

## Figures and Tables

**Table 1:** Pathways, targets, and mechanisms of ginsenosides in regulating metabolic reprogramming.

Ginsenoside	Pathways in Metabolic Reprogramming	Core Metabolic Targets and Molecular Mechanisms	Metabolic Phenotype/Physiological Effects	References
Rg1	AMPK/PI3K-Akt/Nrf2 Pathways	Activate Nrf2-ARE pathway; activate PI3K/Akt/Nrf2 pathway; enhance SOD levels.	Reduce ROS and MDA levels, alleviate oxidative stress and lipid peroxidation.	[64,65,71]
Rg3	GLP-1 Secretion and Insulin Signal Regulation	Increased secretion of GLP-1; increased release of insulin.	Regulate glucose homeostasis.	[83]
Re	Mitochondrial Energy Metabolism/PI3K-Akt Pathway	The PI3K/Akt pathway enhances the activity of mitochondrial complex I.	Rescue dopaminergic neuronal energy metabolism dysfunction to alleviate neurodegeneration in PD models.	[31]
Rg3	Mitochondrial Phosphatidylserine Metabolism and Homeostasis	Regulate mitochondrial phosphatidylserine levels.	Improve motor function in PD model mice and protect mitochondrial homeostasis.	[84]
Rb1	PI3K-Akt Pathway	Activate PI3K/Akt pathway, downregulate LC3-II and Beclin-1.	Restore neuronal homeostasis after ischemia/reperfusion injury and reduce cell death.	[30]
RGs	PKA/CREB Pathway	Activate PKA/CREB pathway.	Reverse the obesity phenotype, improve lipid metabolism and energy expenditure.	[85]
Rg1	HPA Axis Metabolic Regulation	Upregulate glucocorticoid receptors; downregulate FKBP51 expression; enhance negative feedback regulation of the HPA axis.	Alleviate chronic stress-induced metabolic disorders (such as impaired glucose tolerance) and depressive-like behaviors.	[37,86]
Rb1	Regulation of Glutamate Metabolism	Increased GLT-1 in astrocytes.	Inhibit glutamate excitotoxicity, maintain neurotransmitter metabolic balance, and protect dopaminergic neurons.	[28]

**Table 2:** Key examples of relevant ginsenoside clinical trials and their corresponding registration/protocol IDs.

Disease/Condition	Ginsenoside(s)	Registration/Protocol IDs	Description/Phase	References
Advanced Gastric Cancer	Ginsenoside Rg3	NCT01757366	Safety and efficacy of Rg3 combined with first-line chemotherapy.	[89,90]
Breast Cancer (Biomarker)	Ginsenosides (American ginseng)	SCI 07-001.1, IND 79586	Biomarker response in breast cancer patients.	[91]
Stroke Prevention (Atherosclerosis)	Ginsenosides (Korean red ginseng)	NCT02796664, KGC2016-26	Preventive effects on atherosclerosis and ischemic stroke.	[92]
Acute Ischemic Stroke	Ginsenoside Rd	NCT00815763	Efficacy/safety; Phase III, multicenter, China.	[98]
Diabetic Wounds	Ginsenoside Rg1	ChiCTR2200055194	Ginsenoside Rg1 combined with reduced glutathione for the treatment of diabetic wounds.	[99]
Early Chronic Kidney Disease	Ginsenoside Rb1	Clinical trial, but no registration number provided.	Rb1 improves renal function.	[100]
Septic Acute Lung Injury	Ginsenosides	Clinical trial, but no registration number provided.	Synergistic effect with ulinastatin.	[101]

**Table 3:** Advantages and limitations of current ginsenoside drug delivery systems.

Delivery System Classification	Key Advantages	Limitations	References
Nanoemulsion/microemulsion	Thermodynamic or kinetic stability; Nasal administration bypasses the blood–brain barrier.	Use of high-concentration surfactants; Lack of long-term toxicity data.	[3,128]
Liposome	Simultaneously encapsulates hydrophilic/hydrophobic drugs, protecting them from gastric acid and enzymatic degradation.	Poor colloidal stability, prone to aggregation; Phospholipids are susceptible to oxidative hydrolysis; Limited drug loading capacity.	[128]
Polymer nanoparticles	Precise control over drug loading and release; Biodegradability; Active targeting achievable through ligand modification.	The sudden release effect exists; Degradation products may induce inflammatory reactions; Difficulty in controlling batch-to-batch reproducibility.	[3,136]
Solid lipid nanoparticles/nanostructured lipid carriers	High encapsulation efficiency.	Limited drug loading capacity; Drug precipitation caused by crystal transformation.	[3]
Microemulsion delivery system	Improve the absorption of fat-soluble drugs.	High-concentration organic solvents and surfactants; Formulation adjustments required.	[128]
Traditional tablets/capsules	Simple manufacturing process; High patient compliance.	Reasons for inapplicability: Extremely low oral bioavailability; Lack of protection against P-glycoprotein efflux and gastrointestinal degradation.	[132]
Ordinary hydrogel	Local administration; Sustained release, with locally high concentration.	Reasons for inapplicability: Difficulty penetrating skin or mucosal barriers; Unstable gastric environment; Drug burst release; Susceptibility to rupture in swollen state mechanisms, leading to concentration fluctuations due to drug burst release; poor mechanical strength in swollen state, prone to rupture.	[133,134,135]

## Data Availability

No new data were created or analyzed in this study. Data sharing is not applicable to this article.

## Abbreviations

The following abbreviations are used in this manuscript:

ACh	acetylcholine
AChE	acetylcholinesterase
AI	artificial intelligence
AKT	protein kinase B
AMPK	adenosine monophosphate-activated protein kinase
ATF4	activating transcription factor 4
ara(p)	arabinopyranose
ara(f)	arabinofuranose
BDNF	brain-derived neurotrophic factor
cAMP	cyclic adenosine monophosphate
CREB	cAMP response element binding protein
CYP450	cytochrome P450s
GLT-1	glutamate transporter-1
GST	glutathione S-transferase
glc	glucose
HaCaT	human immortalized epidermal cells
HHP	high hydrostatic pressure
HO-1	heme oxygenase-1
HPA	hypothalamic–pituitary–adrenal
HPG	hypothalamic–pituitary–gonadal axis
HP-MAE	high-pressure microwave-assisted extraction
IL	interleukin
JAK2	janus kinase 2
LC3	microtubule-associated protein 1 light chain 3
LWGs	Low-polarity ginsenosides
MAPK	mitogen-activated protein kinase
MDA	malondialdehyde
MMIP	magnetic molecularly imprinted polymer
MMP-1	matrix metalloproteinase-1
MMPs	matrix metalloproteinases
MSNPs	mesoporous silica nanoparticles
NF-κB	nuclear factor kappa-B
NMDA	N-methyl-D-aspartic acid receptor
NR1	NMDA receptor protein
Nrf2	nuclear factor eyrthroid 2-related factor 2
NSCLC	non-small-cell lung cancer
OA	oleanolic acid
OGD	oxygen-glucose deprivation
OT	ocotillol
PCNA	proliferating cell nuclear antigen
PD	Parkinson’s disease
PEF	pulsed electric field extraction
P-gp	P-glycoprotein
PI3K	phosphatidylinositol 3-kinase
PPD	protopanaxadiol
PPT	protopanaxatriol
RGs	rare ginsenosides
ROS	reactive oxygen species
rha	rhamnose
SFE	supercritical fluid extraction
SOD	superoxide dismutase
TACE	transcatheter arterial chemoembolization
T-ALL	T-Cell Acute Lymphoblastic Leukemia
TNF-α	tumor necrosis factor-α
TrkB	tyrosine kinase receptor B
TrkB shRNA	tyrosine kinase receptor B short hairpin RNA
UPE	ultrahigh-pressure extraction
UV	Ultraviolet Rays
VEGF	vascular endothelial growth factor
xyl	xylose
